# Performance characteristics of STANDARD Q Filariasis Antigen test (QFAT) to detect filarial antigens of *Wuchereria bancrofti* in the field

**DOI:** 10.1371/journal.pntd.0012538

**Published:** 2024-09-23

**Authors:** Raja Jeyapal Dinesh, Kaliannagounder Krishnamoorthy, Rajendran Dhanalakshmi, Priskilla Johnson Jency, Palappurath Maliyakkal Azad, Sugeerappa Laxmanappa Hoti, Ashwani Kumar

**Affiliations:** 1 ICMR-Vector Control Research Centre, Puducherry, India; 2 Saveetha Institute of Medical and Technical Sciences (SIMATS), Saveetha University, Chennai, India; TOBB Economics and Technology University Faculty of Medicine: TOBB Ekonomi ve Teknoloji Universitesi Tip Fakultesi, TÜRKIYE

## Abstract

**Background:**

Mapping, monitoring, and evaluation of the Global Programme to Eliminate Lymphatic Filariasis (GPELF) rely on high-throughput diagnostics. While the WHO-recommended Filariasis Test Strip (FTS) is widely used to evaluate the programme, its use is limited by some technical and operational issues. We evaluated the performance characteristics of Q Filariasis Antigen Test (QFAT) compared to FTS for detecting *Wuchereria bancrofti* filarial antigen in the field.

**Methods:**

The QFAT and FTS kits were tested simultaneously for circulating filarial antigen (CFA) during an epidemiological monitoring survey (EMS) in two blocks of a filariasis endemic district in Karnataka, India, as a part of evaluation of the filariasis elimination programme with three drugs (Ivermectin, Diethylcarbamazine, and Albendazole-IDA). Blocks are considered as the evaluation unit as per the revised national guidelines. Two sentinel and one random site from each block with a sample size of 300 individuals aged ≥20 years were selected for the EMS. The field evaluation of the new kit was carried out in the four sentinel sites. Positive tests with either FTS or QFAT or both were tested for microfilaria (Mf) using night blood samples. The performance of the tests was compared in terms of sensitivity, specificity, and predictive values. The percentage agreement between the tests was verified using Cohen’s kappa statistics (k), with a P value of less than 0.05 indicating statistical significance.

**Findings:**

A total of 1227 individuals were tested for CFA using both the QFAT and FTS tests. The QFAT test detected 299 positive individuals at the end of 10 minutes, while the FTS detected 310 positives. The QFAT showed high sensitivity (95.5%), specificity (99.7%), positive predictive value (99.0%), and negative predictive value (98.5%), and the results were in near perfect agreement with those of the FTS (k = 0.97, P <0.001) when the results were read at 10 minutes. There were 17 discordant results that were positive according to either one of the tests. Both antigen tests were positive for all 68 microfilaria-positive samples. None of the QFAT tests were invalid, while three FTS tests were invalid due to non-flow on the test pad. There was no cross-reactivity of the QFAT with *Brugia malayi*-positive samples (n = 5). The feedback from the technicians indicates that QFAT tests were easier to perform compared to FTS in the field.

**Conclusions:**

The Q filariasis antigen test is a promising tool for detecting the *Wuchereria bancrofti* antigen. The kits may be further validated for the review of Diagnostic Technical Advisory Group for Neglected Tropical Diseases (DTAG), to be recommended for the Global Programme to Eliminate Lymphatic Filariasis (GPELF).

## Author summary

Globally, lymphatic filariasis (LF) is targeted for elimination by 2030. Since its inception in 2000 until 2018, the Global Programme to Eliminate Lymphatic Filariasis (GPELF) has witnessed a 74% decline in people infected with LF. As we progress towards the elimination goal, more sensitive diagnostic tools are necessary for inclusion in the GPELF diagnostic portfolio. At present, the WHO recommended filariasis test strip (FTS) is extensively used to evaluate the LF elimination programme worldwide. However, FTS is documented to have certain drawbacks. As a part of a multicentric study, we evaluated the field performance of a new diagnostic kit that detects *Wuchereria bancrofti* filarial antigenemia. The new kit called the Q Filariasis Antigen Test (QFAT), field tested in an LF endemic area in India, had a sensitivity of 95.5% and a specificity of 99.7% compared to FTS, and both tests (FTS and QFAT) detected all *W*. *bancrofti* microfilaria-positive (100%) individuals. The Cohen’s kappa of 0.97 suggested near-perfect agreement between the test results. The test was easy to perform and interpret in the field and required only 20 μL of capillary blood, and no invalid tests were reported. This study documented that the QFAT has comparable performance characteristics with the currently used FTS in field settings, suggesting it to be a promising diagnostic tool. The pooled analysis of performance characteristics from other geographic areas will enable a decision on its inclusion in the diagnostic portfolio of GPELF.

## Introduction

Lymphatic filariasis (LF) is a neglected tropical disease and is targeted for elimination as a public health problem by 2030 [1,2]. In 2021, 882.5 million population residing in 44 endemic countries were at risk, thus requiring preventive chemotherapy [3]. The Global Programme to Eliminate Lymphatic Filariasis (GPELF) has made significant progress over the past decade, as evidenced by the robust monitoring and evaluation framework, which adds both rigour and standardization to the decision-making process [4,5]. So far, 19 countries have been certified to have eliminated LF as a public health problem, while 11 other countries are under surveillance for certification [4]. Monitoring and evaluation of the LF elimination programme requires highly efficient surveillance systems and diagnostic tools that are accurate, reliable, and appropriate to support programmatic decisions [6].

The WHO recommends the use of Bioline Filariasis Test Strips (FTS), manufactured by Abbott. It detects circulating filarial antigen (CFA) during various steps in the GPELF strategy [7–9]. Although FTS has been commonly used, many disadvantages have been identified. The test requires 75 μL of capillary blood collected by a finger-prick, and issues with proper flow of serum on the test pad have been reported [10]. Furthermore, there is no protective casing for narrow and lightweight test strips, which poses logistical challenges in the field [9,10]. The test strips need to be secured with tape or some suitable adhesive to minimize their movement [10]. Finally, reliance on a single manufacturer poses procurement challenges for global and national requirements [10,11]. A new rapid test that requires only 20 μL of capillary blood to detect *Wuchereria bancrofti* circulating filarial antigen (CFA), the STANDARD Q Filariasis Antigen Test (QFAT), has been developed as a simple card format by SD Biosensor, South Korea. The performance of the new kit in laboratory settings yielded a sensitivity of 92% and a specificity of 98%, and it is reported to be an appropriate test for use in the LF elimination programme [10]. However, the new kit is yet to be evaluated for its performance under field conditions.

This study evaluated the performance characteristics of the QFAT compared to those of the Bioline Filariasis Test Strip (FTS) in detecting filarial infection under field settings in India. The secondary objectives were (i) to determine the feasibility and ease of use of the QFAT in field settings; (ii) to determine the rate of invalid or indeterminate results of the QFAT and FTS; and (iii) to determine the cross-reactivity of the QFAT with *B*. *malayi* microfilaria (Mf) positive individuals. The results of this study will enable the WHO to determine whether the QFAT is a suitable tool for inclusion in the GPELF diagnostic portfolio. This is being done to inform the WHO and the National Centre for Vector Borne Diseases Control (NCVBDC, India) that an alternative diagnostic tool is available for monitoring and evaluation of LF programmes. The ICMR-Vector Control Research Centre (ICMR-VCRC) at Puducherry, a WHO-collaborating centre for research and training on lymphatic filariasis and integrated vector management, conducted the field validation of this new diagnostic kit in sites selected for evaluation of the LF elimination programme (using FTS) as a support to the WHO.

## Methods

### Ethical statement

The study was approved by the Institution Human Ethics Committee (IHEC-0122/N/J, dated 21^st^ July, 2022) and the Health Ministry’s Screening Committee (HMSC, April 2023). Necessary approvals were also obtained from the National Centre for Vector Borne Diseases Control (NCVBDC), Karnataka state, and Bidar district programme. The research team explained the purpose of the study in the local language (Kannada) and obtained written informed consent from all the participants. All procedures followed the pertinent rules and principles of the Declaration of Helsinki. Individuals found positive with FTS were treated as per the national guidelines by the district health team.

### Study design and participants

The QFAT was evaluated in the Bidar district after two rounds of mass drug administration (MDA) with three drugs (Ivermectin, Diethylcarbamazine, and Albendazole-IDA). Health blocks (subdistricts) with populations not exceeding 0.5 million are the evaluation units according to the revised national guidelines for evaluating filariasis elimination programme in India [12]. The block-level strategy recommends the selection of two sentinel sites (wards or villages) and one random site for the Mf survey. From each site, 300 individuals above the age of 19 years should be tested either by night blood sample for Mf or by FTS test, followed by a night blood sample from CFA-positive individuals. Once the Mf prevalence is less than 1% at each of the three sites, it will be considered for the IDA impact survey. The new diagnostic test, the QFAT, was tested in parallel with the FTS as a part of the epidemiological monitoring survey (EMS) activity of the programme from June–July 2023.

### Study area

**Fig 1:**
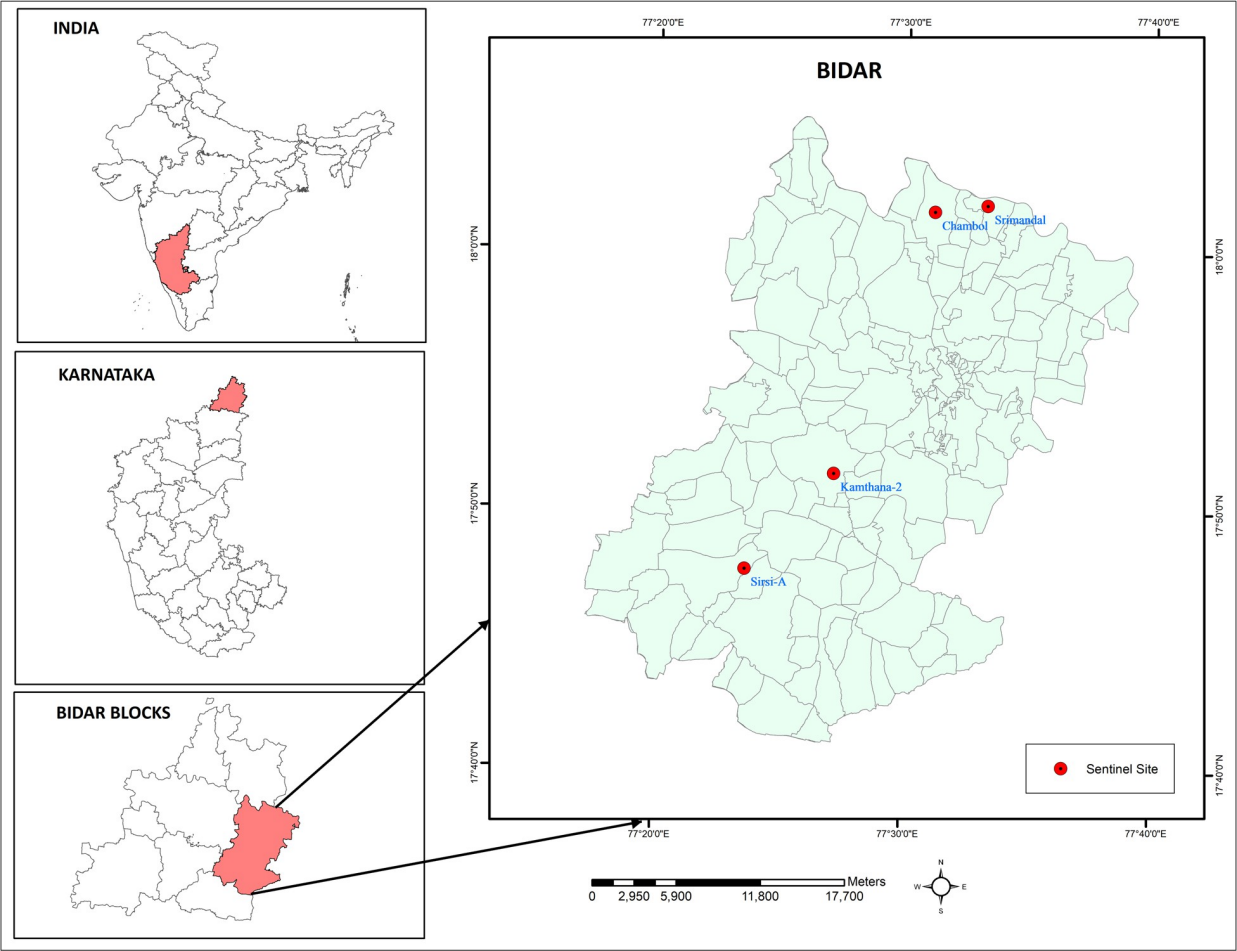
Map showing the sentinel sites selected for the study in Bidar district of Karnataka, India. Base layer of map: https://onlinemaps.surveyofindia.gov.in. License information: https://onlinemaps.surveyofindia.gov.in/GeospatialGuidelines.aspx. ArcGIS Software: https://www.arcgis.com.

The Bidar district is located in the northeastern part of Karnataka, bordering the states of Maharashtra and Telangana in India. The district is endemic for *Culex quinquefasciatus*, which transmits bancroftian filariasis and has undergone more than six rounds of MDA with two drugs (Diethylcarbamazine, and Albendazole) since 2004. MDA with IDA (Ivermectin, Diethylcarbamazine, and Albendazole) was introduced in 2021 in all nine blocks of the Bidar district, and two rounds of annual MDA were completed by 2023. Block-level assessment by the annual Mf survey reported Mf <1% in all sentinel and random sites, thus qualifying for the use of an epidemiological monitoring survey [12]. Two blocks (Bidar block A and Bidar block B) with a high prevalence of filarial lymphoedema cases were selected purposively in consultation with the district health authorities. As per the guidelines, two sentinel sites and one random site were selected, and the field evaluation was carried out only at two sentinel sites in each block [12]. For the programmatic decision, the results of all three sites in each block were considered. Chambol and Srimandal in Bidar block A and Kamthana 2 and Sirsi A in Bidar block B were the sentinel sites selected for this study (Fig 1).

### Target population

All consenting individuals aged ≥20 years of either gender, regardless of their participation in previous annual MDA rounds at the selected sites in the Bidar district, Karnataka, India.

## Procedures

### Sampling and data collection plan

**Fig 2:**
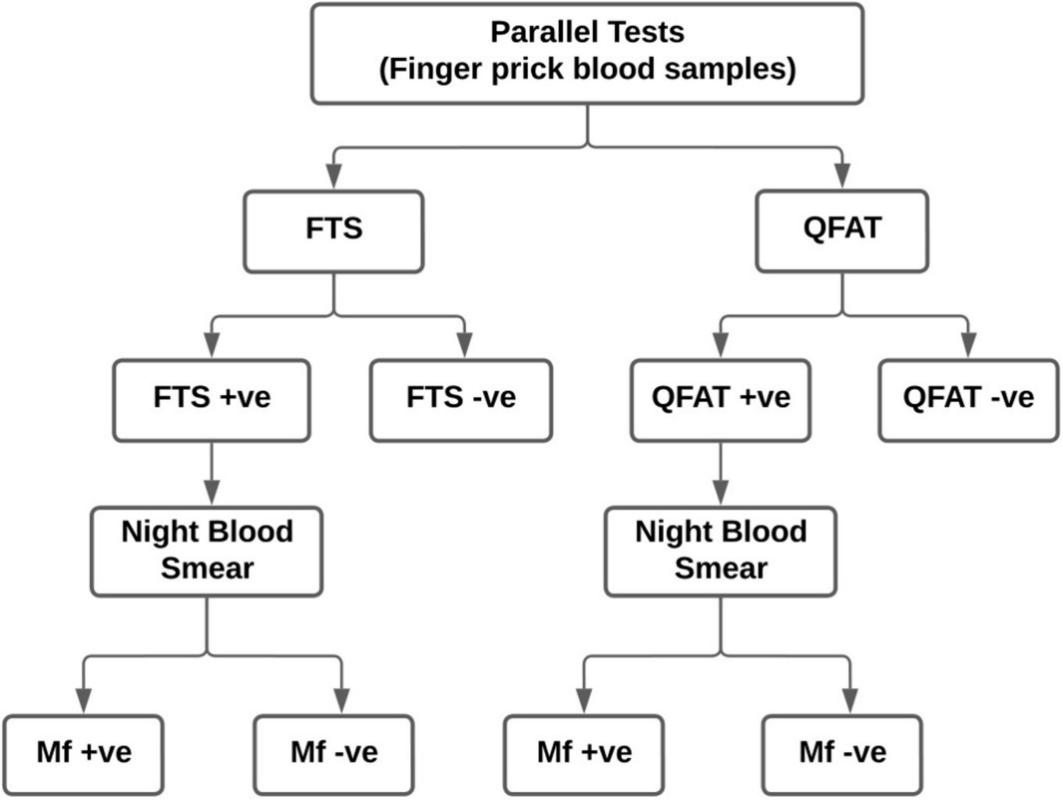
Flowchart showing the sampling plan at each site in Bidar, Karnataka, India.

The survey was conducted during the day in camp mode instead of visiting every household identified by systematic sampling, owing to time constraints. The district health staff visited the houses (convenience sampling) and mobilized them to report to the campsite to test for filarial infection. The purpose of the study was explained to all eligible individuals visiting the camp. It was left to the discretion of the individual to decide whether to participate or not in the survey. The voluntarily consenting participants were enrolled through the use of a semi-structured questionnaire that collected details on socio-demographics. Testing of individuals was done while the survey was conducted as a part of the Epidemiological Monitoring Survey. Approximately 95 μl of capillary blood was collected using micropipettes by the finger prick method for simultaneous testing of FTS (75 μl) and QFAT (20 μl). Individuals who were positive for FTS, QFAT, or both were visited again after 21:00 hours the same day to collect night blood smears (60 μl) for Mf. During this process, the QFAT was once again repeated at night with samples obtained from these individuals (QFAT positives during the day). Repeated house visits were made to sample the absentees of CFA-positive individuals. Finger-prick blood samples were also collected from 47 FTS-positive individuals (from three sites) into heparinized microtainers and later tested with QFAT kits. The scheme for screening individuals is shown in Fig 2. A unique barcode was allotted to every enrolled individual, and the same barcode was affixed to the enrolment form, consent form, FTS kit, QFAT kit, blood slide, and microtainer. Additionally, stored serum samples collected from five *B*. *malayi* Mf-positive individuals from the Balasore district, Orissa, were used to evaluate the cross-reactivity of the QFAT test kit.

### Field and laboratory tests

#### Bioline Filariasis test strip

The FTS kits were provided by the district programme, and were previously donated by the WHO for the evaluation of MDA. The kits were used according to the manufacturer’s instructions. About 75 μl of whole blood was placed on the FTS test pad using a micropipette, and the results were read at exactly 10 minutes by the FTS test reader. The scoring of the positive test was performed based on the colour intensity of the test line with respect to the control line as follows: strongly positive 3+ (test line darker than the control line), moderately positive 2+ (test line as strong as the control line), and weakly positive 1+ (test line lighter than the control line). Any invalid test with FTS was repeated once.

#### STANDARD Q Filariasis Ag test

The kits were supplied by the WHO and were tested simultaneously in the field according to the manufacturer’s instructions. About 20 μl of capillary blood was placed on the test pad using a micropipette, and two drops of chase buffer were added to the well. The manufacturer instructions required to read the test results between 10–20 minutes after sample application. In this study, the results were read exactly at 10 minutes and 20 minutes to see if there was any difference between testing at the beginning and the end of the allowed interval. Teams were instructed to repeat any invalid tests once. The scoring of the positive test was performed based on the colour intensity of the test line, similar to FTS. The QFAT tests were repeated once again at the time of night blood sample collection for those who tested QFAT positive during the day.

#### Mf

Night blood smears were collected for the detection of microfilariae of *W*. *bancrofti* from individuals positive for either FTS and/or QFAT (Fig 2). Approximately 60 μl of finger-prick blood was collected using a capillary tube, and three parallel lines of each 20 μl (smears) were made on the slide [13]. The slides were air-dried, packed, and later stained with Giemsa following standard processing procedures. One technician was assigned to process the blood slides in the field. The blood smear examination was independently performed via microscopy by two trained technical staff members, and the results were compared. All positive slides and at least 10% of the negative slides were cross-examined by senior technical staff.

#### Field team composition

Two field teams were formed and trained to carry out the field surveys. Each team comprised 8 members: (i) an interviewer, (ii) a smartphone handler, (iii) a supply manager, (iv) a phlebotomist, (v) a FTS test preparer, (vi) a FTS test reader, (vii) a QFAT test preparer, and (viii) a QFAT reader. Additionally, each team was supervised in the field by 1–2 staff members from the ICMR-VCRC to ensure quality. Both FTS and QFAT tests were performed simultaneously using a sample obtained from a single finger-prick, and the results were read by two independent test readers. All the test results were reconfirmed by the supervisory staff in each team before being entered into the proforma. However, it was difficult to blind the entire process of testing and reading of results as both the tests were carried out simultaneously in the field and participants were waiting for their test results. The same team collected night blood samples from FTS and/or QFAT positive subjects for Mf on the same night and, if not available, the next night.

### Statistical analysis

#### Sample size

Following recent Epidemiological Monitoring Survey (EMS) guidelines of the WHO, a minimum of 300 individuals aged ≥20 years were screened from each site. Testing of the diagnostics was performed with a minimum of 1200 individuals from the four sentinel sites in the two identified blocks (Bidar blocks A and B) in the Bidar district, Karnataka.

#### Data management and analysis

Data were simultaneously collected in a semi-structured proforma (responses were handwritten) and captured electronically using Kobo Toolbox software on smartphones. The data captured on the smartphones were cross-verified from handwritten proforma. Further analysis was carried out using SPSS v.21.0 and STATA v.17.0. The details of the socio-demographics, such as age and sex, are expressed as either the mean (SD) or proportion, whichever was applicable. The performance characteristics of the new diagnostic kit (primary outcome) were obtained by comparing the results of the QFAT with those of the FTS (gold standard test). The sensitivity, specificity, and predictive values (positive and negative tests) with 95% confidence intervals (95% CIs) were calculated. The agreement between the two tests was expressed as a percentage, and Cohen’s kappa (k) statistics were interpreted as follows: k value ≤ 0: no agreement; 0.01–0.20: none to slight; 0.21–0.40: fair agreement; 0.41–0.60: moderate agreement; 0.61–0.80: substantial; and 0.81–1.00: near perfect agreement. The area under the receiver operating characteristic curve (AUC) with a 95% CI was calculated to determine the overall performance. An AUC > 0.9 was considered to indicate outstanding performance.

## Results

A total of 1227 individuals aged ≥ 20 years (mean age 43.7±16.6 years; range 20–85 years), comprising 60.4% females and 39.6% males, were simultaneously screened for CFA using FTS and QFAT kits from the four selected sentinel sites. A total of 299 (24.4%) and 310 (25.3%) samples tested positive for QFAT and FTS at 10 minutes, respectively.

### Performance characteristics

Compared with those of the FTS test, the sensitivity, specificity, and predictive values (positive and negative) of the QFAT were greater than 95%, 99%, and 98%, respectively (Table 1).

**Table 1:** Performance characteristics of the QFAT in comparison with the FTS results at 10 minutes.

**Diagnostic test**	**Test results**	**FTS Positive**	**FTS Negative**	**Total**	**Sensitivity = 95.5% (94.3–96.7)** **Specificity = 99.7% (99.4–99.9)** **PPV = 99.0% (98.4–99.6)** **NPV = 98.5% (97.8–99.2)** **AUC = 0.985 (0.975–0.995)** **% agreement = 98.9%** **k value = 0.970 (P <0.001)**
**QFAT test** **(at 10 min.)**	Positive	296	3	299	
	Negative	14	914	928	
	Total	310	917	1227	

In parenthesis—95% confidence intervals, PPV- positive predictive value, NPV- negative predictive value

AUC- area under the curve, k value- kappa statistics

### Concordance between tests

The QFAT results (positive or negative for CFA) were 98.9% in agreement with the FTS results, and the kappa statistics (k value 0.97, P<0.001) suggest near-perfect agreement between the two tests (Table 1).

### Discordance between tests

There were 17 discordant results, that were positive by either one of the tests. Fourteen samples that tested FTS positive were QFAT negative and three samples that tested negative for FTS, tested positive for QFAT at the end of 10 minutes (Table 1). However, all 17 discordant results were Mf negative on slide examination.

### Performance characteristics in terms of colour intensity scores (FTS vs. QFAT)

In terms of scores based on the intensity of colour of the test line, there was only moderate agreement (kappa statistics ≈0.52) between the two tests. None of the strong positive (3+) FTS scored 3+ by the QFAT test. All 3+ FTS-positive samples were positive by QFAT but all of them scored either 2+ or 1+ (Table 2).

**Table 2:** Performance characteristics (FTS vs QFAT) in terms of intensity scores at 10 minutes.

Diagnostic test		FTS results (at 10 min.) in relation to test-line colour intensity (% out of “n” of the column)				Total	k value (P value)
		0	1+	2+	3+		
**QFAT score** **(read at 10 min.)**	**0**	914 (99.7)	11 (16.2)	2 (1.6)	1 (0.9)	928 (75.6)	0.954 (<0.001)
	**1+**	2 (0.2)	56 (82.4)	116 (89.9)	61 (54.0)	235 (19.2)	0.310 (<0.001)
	**2+**	1 (0.1)	1 (1.5)	11 (8.5)	51 (45.1)	64 (5.2)	0.048 (0.040)
	**3+**	0 (0.0)	0 (0.0)	0 (0.0)	0 (0.0)	0 (0.0)	-
	**Total**	917	68	129	113	1227	0.521 (<0.001)

### Performance characteristics of QFAT and FTS for detecting *W*. *bancrofti* Mf

Night blood smears were collected from a total of 294 individuals who were positive for either FTS or QFAT, or both. On slide examination, 68 individuals were positive for *W*.*bancrofti*, with Mf counts ranging from 1 to 242 per 60 μl of capillary blood. All 68 Mf-positive individuals also tested positive by both FTS and QFAT, showing 100% concordance.

### Invalid tests

There were three invalid tests with FTS due to the non-flow of serum on the test pad. All three tests were repeated once, and the results were included in the final analysis. Notably, there were no invalid tests with the QFAT.

### QFAT test results read at 20 minutes

The QFAT test results were also read at 20 minutes and compared with the FTS results that were read at 10 minutes (Table 3). The sensitivity of QFAT increased by 2.2% when the results were read at 20 minutes. However, 14 test results were discordant.

**Table 3:** Performance characteristics of the QFAT (20 min.) in comparison with the FTS results (10 min).

**Diagnostic test**	**Test results**	**FTS Positive**	**FTS Negative**	**Total**	**Sensitivity = 97.7% (96.9–98.6)** **Specificity = 99.2% (98.7–99.7)** **PPV = 97.7% (96.9–98.5)** **NPV = 99.2% (98.9–99.7)** **AUC = 0.976 (0.962–0.989)** **% agreement = 98.6%** **k value = 0.963 (P <0.001)**
**QFAT test** **(read at 20 min.)**	**Positive**	303	7	310	
	**Negative**	7	910	917	
	**Total**	310	917	1227	

In parenthesis—95% confidence intervals, PPV- positive predictive value, NPV- negative predictive value

AUC- area under the curve, k value- kappa statistics

### Repeat QFAT test results

A total of 264 QFAT positive tests (during the day) were repeated at the time of night blood sample collection for Mf. When the QFAT results were read at 10 minutes, there were 31 tests that were positive during the day but tested negative on repeating the test at night. All 31 (11.7%) discordant tests were negative for microfilaria. The number of discordant results reduced to six (2.3%) when the QFAT was read again at 20 minutes.

### Cross-reactivity of QFAT with *B*. *malayi* Mf-positive samples

The cross-reactivity of QFAT with *B*. *malayi* was tested with five stored serum samples obtained from *B*. *malayi* positive individuals from the endemic district of Balasore in Odisha State, India, and none of the samples tested positive for QFAT.

### Ease-of-use of the QFAT kits

The fifteen trained technical staff members, involved in field testing agreed that the QFAT kits were easy to use in the field and required less capillary blood (20 μl) than the FTS kits (75 μl). The test procedure was easy to perform, except for the extra step of adding the chase buffer. The control and test lines were clear and easy to read; however, there were chances of missing mild positives (1+) at 10 minutes by novice test readers. Although the manual supplied by the manufacturer provided clear instructions, there was no clarity on the exact time needed to read the results (10 or 20 minutes).

### Samples in heparin-coated microtainer tubes

Capillary blood samples (100 μl) were collected in heparin-coated microtainers from 47 FTS-positive individuals from three sites and tested with QFAT. Comparison of QFAT test results using directly applied whole blood showed 68.1% agreement (heparinized vs. direct samples), k = 0.198 (P = 0.02) at 10 minutes. The percentage agreement was much greater at 93.6%, k = 0.636 (P<0.001), when the results of heparinized and whole blood were read at 20 minutes.

## Discussion

The progress of the GPELF is monitored by screening residents of communities under the MDA for the presence of microfilariae or the CFA for *W*. *bancrofti* [6]. A demonstration of the prevalence of Mf < 1% or a CFA < 2% is an indication for interruption of transmission and warrants stopping further MDA rounds. The WHO recommended filariasis test strips (FTS), manufactured by Abbott, for all areas endemic to *W*. *bancrofti*. The FTS, which measures CFA, is commonly used during all steps of the GPELF strategy, namely, mapping, impact assessment, pre-TAS, TAS, and post-validation surveillance [13]. Although the FTS is widely used by LF programmes across several countries, it has some technical disadvantages [7–10]. In the quest for newer tools to support programmatic decisions, a new filarial antigen-based rapid diagnostic test for *W*. *bancrofti*, the QFAT, showed promising results during its laboratory evaluation [10]. The present study compares the performance characteristics of the QFAT with those of the FTS under field conditions. These field trials are necessary not only to assess the performance characteristics in different prevalence areas but also to evaluate the ease of test use and ensure achievement of satisfactory test performance in the hands of teams working in the field [14].

The QFAT results were in almost perfect agreement with the FTS results at 10 minutes (98.9% agreement, k value 0.97) for detecting *W*. *bancrofti* circulating filarial antigen. Laboratory validation of the QFAT using stored serum and plasma samples from LF-endemic areas in the Asia-Pacific region revealed slightly lower values of agreement (93.5% agreement, k value 0.87) and a sensitivity and specificity of 92% and 98%, respectively [10]. Field evaluation of QFAT in Samoa reported a similar concordance (98.5%) and excellent agreement (k value 0.96) between QFAT and FTS tests at 10 minutes [11]. The utility of a diagnostic tool is defined by its performance characteristics, and a high sensitivity and specificity are often considered optimal benchmarks [15]. Diagnostic tools with high specificity are desired to measure infection in low-prevalence settings [14]. It may be noted that the sensitivity of the new diagnostic test increased by 2.2% and the specificity remained above 99% when the results were read at 20 minutes. However, the corresponding results of FTS at 20 minutes were not recorded to carry out an ideal comparison.

All 68 Mf positives showed positive reactions (100% concordance) by both tests. Therefore, information on Mf prevalence is more appropriate in the evaluation of IDA-MDA as it is more efficacious in clearing Mf than CFA [16]. A study in American Samoa showed that ICT had 93.8% sensitivity and 100% specificity for identifying FTS-positive persons. For those who were FTS-positive, the study compared ICT sensitivity and specificity between Mf-positive (n = 28) and Mf-negative (n = 72) persons and did not find any statistically significant difference [17].

In the present study, 23.1% (68 of 294 CFA positives) of individuals were microfilaremic in all four sentinel sites. High microfilaremia rates observed in the present survey could be attributed to non-participation in the IDA-MDA rounds. There were 509 (41.5%) individuals who self-reported having never participated in either of the IDA-MDA rounds in the present study. The programme continued with one more round of IDA-MDA in the blocks on 10^th^ August, 2023.

In terms of scores based on the colour intensity of the test line, there was poor to fair agreement between the two tests. None of the QFAT tests produced a strong test line colour (3+), although they mostly identified all CFA positives (including FTS 3+) with lower scores (1+ or 2+) based on the test line colour. This was in concordance with the results of the field evaluation of QFAT at Samoa which reported that none of the samples produced a test line darker than the control line. They argued that stronger control lines produced with QFAT are less prone to misinterpretations and, hence, are more reliable in field settings [11]. A study in the Democratic Republic of the Congo reported a strong correlation between the colour intensity score of FTS and CFA levels in plasma measured by the Og4C3 ELISA [18]. A similar comparison may be useful to explain the colour intensity of the test line.

Upon repeating the QFAT positive tests (during the day) at night, 31 (11.7%) results were discordant when read at 10 minutes and it reduced to six (2.3%) when the results were read again at 20 minutes. However, the QFAT negative tests (during the day) were not repeated at night due to the limited number of kits. This necessitates further evaluation to ascertain the time of reading of QFAT results either at 10 or 20 minutes.

None of the discordant tests were Mf positive. The IDA regimen appears to suppress Mf as this district had undergone two rounds of IDA-MDA following several rounds of DA-MDA (the timing of present survey was over 9 months after the second round of IDA-MDA). Most of the Mf carriers might likely have converted to amicrofilariaemics due to multiple MDA rounds over a long term. Although no technical reasons could be attributed, studies are needed to address this issue.

The QFAT was not reactive with samples positive for *B*. *malayi* Mf, though a small sample of five, may indicate that the test is specific to *W*. *bancrofti*. However, its cross-reactivity with other nematode parasites such as *Loa loa* and *Onchocerciasis* needs to be assessed for its use in other co-endemic areas. These performance characteristics of the QFAT, in addition to its ease of use in the field, suggest that the QFAT could be a potential alternative to the existing FTS for use in LF elimination programmes.

During the laboratory evaluation, Graves et al., 2024 reported that 0.4% and 0.2% of the tests were invalid by QFAT and FTS, respectively [10]. However, in the present study, no invalids were reported with QFAT while the proportion invalid tests with FTS were similar. The field evaluation of QFAT in Samoa also reported no invalids with QFAT but 4.1% with FTS kits [11]. The addition of chase buffer in the QFAT test might facilitate the free flow of blood samples on the test pad. Lateral flow assays are often affected by environmental factors such as temperature and relative humidity and require proper storage under ambient conditions [19]. The absence of an additional buffer step, and no flow of serum on the test pad could be the probable reasons for invalid tests with FTS [8].

The QFAT test results with heparinized blood showed 68% agreement with whole blood sample results when read at 10 minutes, and it increased to 98% when read at 20 minutes. Probably when heparinized blood is used, reading the QFAT result at 20 minutes may be considered. This can be ascertained by conducting studies with a larger sample size.

One of the limitations of this study is that the FTS results were read only at 10 minutes, while the QFAT results were read at 10 minutes and 20 minutes as per the manufacturer’s instructions. Hence, the results of both tests could not be compared at 20 minutes. Additionally, the FTS tests were not repeated at night as only a limited number of kits were provided by the district programme. The QFAT kits were not tested for cross-reactivity with other parasitic infections, such as soil-transmitted helminths (STHs). It was difficult to blind the entire process of testing and reading of results as both the tests were carried out simultaneously in the field using samples taken from the same finger prick and participants were waiting for their test results. The intensity of colour of the test line is in relation to that of the control line in both tests, which constrained any direct comparison of results between the two tests. Evaluation of QFAT kits in different endemic, co-endemic (with other helminthic infections), geographic, and prevalence settings may be useful before being reviewed by the Diagnostic Technical Advisory Group for Neglected Tropical Diseases (DTAG) to recommend its inclusion in the diagnostic portfolio of GPELF.

## Conclusion

The performance characteristics of the STANDARD Q Filariasis Antigen Test (QFAT) in field settings make it a promising diagnostic tool for use in global programme to eliminate lymphatic filariasis.
